# *CNGB3* Missense Variant Causes Recessive Achromatopsia in Original Braunvieh Cattle

**DOI:** 10.3390/ijms222212440

**Published:** 2021-11-18

**Authors:** Irene M. Häfliger, Emma Marchionatti, Michele Stengård, Sonja Wolf-Hofstetter, Julia M. Paris, Joana G. P. Jacinto, Christine Watté, Katrin Voelter, Laurence M. Occelli, András M. Komáromy, Anna Oevermann, Christine Goepfert, Angelica Borgo, Raphaël Roduit, Mirjam Spengeler, Franz R. Seefried, Cord Drögemüller

**Affiliations:** 1 Institute of Genetics, Vetsuisse Faculty, University of Bern, Bremgartenstrasse 109a, 3001 Bern, Switzerland; irene.haefliger@vetsuisse.unibe.ch (I.M.H.); hofstettersonja@hotmail.com (S.W.-H.); julia-1991@hotmail.com (J.M.P.); 2 Clinic for Ruminants, Department of Clinical Veterinary Medicine, Vetsuisse Faculty, University of Bern, Bremgartenstrasse 109a, 3001 Bern, Switzerland; emma.marchionatti@vetsuisse.unibe.ch; 3 Division of Ophthalmology, Department of Clinical Veterinary Medicine, Vetsuisse Faculty, University of Bern, Länggassstrasse 128, 3001 Bern, Switzerland; michele.stengard@vetsuisse.unibe.ch (M.S.); christine.watte@vetsuisse.unibe.ch (C.W.); 4 Department of Veterinary Medical Sciences, University of Bologna, 50 Ozzano Emilia, 40064 Bologna, Italy; joana.goncalves2@studio.unibo.it; 5 Ophthalmology Section, Vetsuisse Faculty, University of Zurich, Winterthurerstrasse 260, 8057 Zurich, Switzerland; kvoelter@vetclinics.uzh.ch; 6 College of Veterinary Medicine, Michigan State University, 736 Wilson Rd., East Lansing, MI 48824, USA; occelli@msu.edu (L.M.O.); komaromy@msu.edu (A.M.K.); 7 Division of Neurological Sciences, Vetsuisse Faculty, University of Bern, Bremgartenstrasse 109a, 3001 Bern, Switzerland; anna.oevermann@vetsuisse.unibe.ch; 8 COMPATH, Institute of Animal Pathology, Vetsuisse Faculty, University of Bern, Länggassstrasse 122, 3001 Bern, Switzerland; christine.goepfert@vetsuisse.unibe.ch; 9 Department of Ophthalmology, University of Lausanne, Jules-Gonin Eye Hospital, Av. de France 15, 1004 Lausanne, Switzerland; angelica.borgo@fa2.ch (A.B.); raphael.roduit@fa2.ch (R.R.); 10 Qualitas AG, Chamerstrasse 56, 6300 Zug, Switzerland; mirjam.spengeler@qualitasag.ch (M.S.); franz.seefried@qualitasag.ch (F.R.S.)

**Keywords:** *Bos taurus*, animal model, day-blindness, retina, development, mendelian genetics, rare disease, precision medicine

## Abstract

Sporadic occurrence of inherited eye disorders has been reported in cattle but so far pathogenic variants were found only for rare forms of cataract but not for retinopathies. The aim of this study was to characterize the phenotype and the genetic aetiology of a recessive form of congenital day-blindness observed in several cases of purebred Original Braunvieh cattle. Electroretinography in an affected calf revealed absent cone-mediated function, whereas the rods continue to function normally. Brain areas involved in vision were morphologically normal. When targeting cones by immunofluorescence, a decrease in cone number and an accumulation of beta subunits of cone cyclic-nucleotide gated channel (CNGB3) in the outer plexiform layer of affected animals was obvious. Achromatopsia is a monogenic Mendelian disease characterized by the loss of cone photoreceptor function resulting in day-blindness, total color-blindness, and decreased central visual acuity. After SNP genotyping and subsequent homozygosity mapping with twelve affected cattle, we performed whole-genome sequencing and variant calling of three cases. We identified a single missense variant in the bovine *CNGB3* gene situated in a ~2.5 Mb homozygous genome region on chromosome 14 shared between all cases. All affected cattle were homozygous carriers of the p.Asp251Asn mutation that was predicted to be deleterious, affecting an evolutionary conserved residue. In conclusion, we have evidence for the occurrence of a breed-specific novel *CNGB3*-related form of recessively inherited achromatopsia in Original Braunvieh cattle which we have designated OH1 showing an allele frequency of the deleterious allele of ~8%. The identification of carriers will enable selection against this inherited disorder. The studied cattle might serve as an animal model to further elucidate the function of *CNGB3* in mammals.

## 1. Introduction

Food animal ophthalmology is a neglected area of veterinary medicine [1,2]. In farm animal practice, conditions such as visual impairment or even blindness, despite having a negative impact on behavior and welfare, are rarely considered, as there is usually no profoundly damaging economic impact on animal production [3].

Sporadic occurrence of inherited eye disorders in livestock species such as cattle has been reported [4,5,6]. However, many affected newborns go unreported or escape surveillance systems [4]. In addition to hereditary reasons, environmental causes such as vitamin A deficiency and bacterial or viral infections must also be taken into account [5,6,7]. Although congenital eye defects are rare, they are important and should be considered because they often follow monogenic recessive inheritance [4,6]. Therefore, such inherited disorders can rapidly gain in prevalence due to the undetected use of carriers, especially in the course of artificial insemination in cattle. So far pathogenic variants were found only for rare breed-specific recessive forms of cataract in Romagnola (OMIA 001936-9913) and Holstein (OMIA 002111-9913) cattle [8,9]. Such findings enable selection against these disorders within the affected populations. Furthermore, the *NID1*-related cataract observed in Romagnola was the first report of a naturally occurring mutation leading to a non-syndromic form of cataract in a mammalian species [8]. Therefore, the genetic study performed in cattle added the affected gene to the list of candidate genes for inherited forms of nuclear cataract in humans, illustrating the impact of studying eye conditions in domestic animals.

Achromatopsia, an inherited retinal disease characterized by the loss of cone photoreceptor function resulting in day-blindness, total color-blindness, and decreased central visual acuity, has not yet been described in cattle. Pathogenic variants in six genes (*CNGA3*, *CNGB3*, *GNAT2*, *ATF6*, *PDE6C*, and *PDE6H*) have been identified in humans with achromatopsia (OMIM 216900) [10]. In sheep, a form of *CNGA3*-related achromatopsia has been characterized (OMIA 001481-9940) [11]. This ovine condition was intensively used for functional restoration of cone function [12], highlighting the biomedical value of such large animal models [13,14] in addition to the direct benefits for animal breeding and animal health.

The aim of this study was to characterize the phenotype and the underlying causative genetic defect for this presumably new form of congenital day-blindness observed in several cases of purebred Original Braunvieh cattle from Switzerland.

## 2. Results

### 2.1. Clinical Description

Initially, in 2017, a four-month-old calf (case 2) was presented to the University of Zurich for evaluation of suspected day blindness. Ocular examination revealed bilaterally absent menace responses and dazzle reflexes. Pupillary responses were positive. Chromatic light stimulation (Melan) revealed bilaterally minimal dazzle reflexes with red and blue light, pupillary responses were normal with blue and reduced with red light stimulation. The remainder of the ocular exam (slit-lamp biomicroscopy, indirect and direct ophthalmoscopy, intraocular pressure) was within normal limits with the optic nerve head showing a deep physiologic cup. The calf was able to complete an obstacle course in dim light, but not in bright light.

In 2020, a 5.5-month-old calf (case 12) was presented to the University of Bern for evaluation of a suspected vision disturbance. The farmer noted that the calf was hesitant to walk and often collided with obstacles in its environment, especially when separated from the mother (Video S1). Observation of the calf revealed extremely poor navigation of large and small objects in the examination hall in ambient room light. Navigation of obstacles and recognition of large high-contrast obstacles after room lights were turned off was slightly improved. Ophthalmic examination revealed absent menace response in both eyes (OU) in dim, ambient and bright (outdoor) light conditions. The direct and consensual pupillary light responses as well as dazzle reflexes to white light were reduced in both eyes. A slightly reduced pupillary light response was noted with bright blue light stimulation and severely reduced to bright red light stimulation. Slit-lamp biomicroscopic examination of the eyelids, conjunctiva, cornea, anterior chamber, lens and anterior vitreous was normal in both eyes with the exception of mild mydriasis OU. The pupils were symmetrical OU. Fluorescein staining of the cornea was negative OU. Indirect ophthalmoscopy revealed normal appearance to the optic nerve, tapetal and non-tapetal retina, and retinal vessels bilaterally. Intraocular pressure was measured within normal limits (11 mmHg OD, 14 mmHg OS).

**Figure 1:**
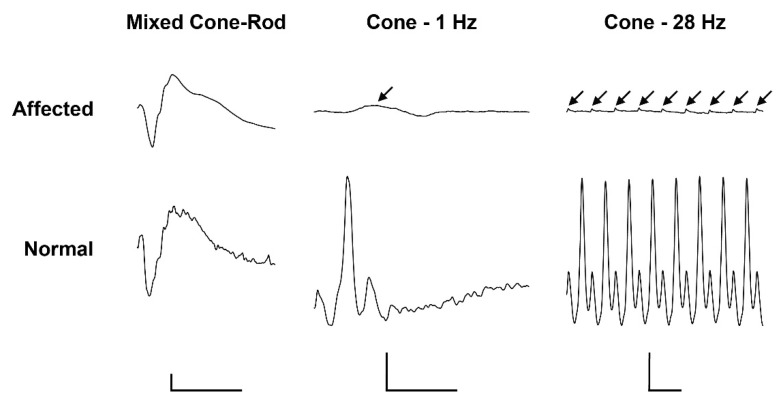
Electroretinogramm (ERG) of a 5.5-month old achromatopsia-affected Original Braunvieh (case 12) and a seven-month old Hereford control cattle. Dark-adaptation responses in representative normal and affected calves. While the dark-adapted mixed cone-rod responses were comparable between the two animals, the light-adapted single cone (1 Hz) and cone flicker (28 Hz) responses were severely reduced (arrows). Calibration bars: vertical = 100 µV, horizontal = 50 ms.

Electroretinography in the affected calf revealed severely reduced light-adapted single cone and cone flicker responses, while the dark-adapted mixed cone-rod response was considered normal (Figure 1). Combined with the behavioral observations and clinical findings, the ERGs support an achromatopsia diagnosis based on the specific loss of cone-mediated retinal function without any retinal degenerative changes.

### 2.2. Pathological Phenotype

During necropsy of case 12 no gross lesions were detectable and brain areas involved in vision were morphologically normal.

**Figure 2:**
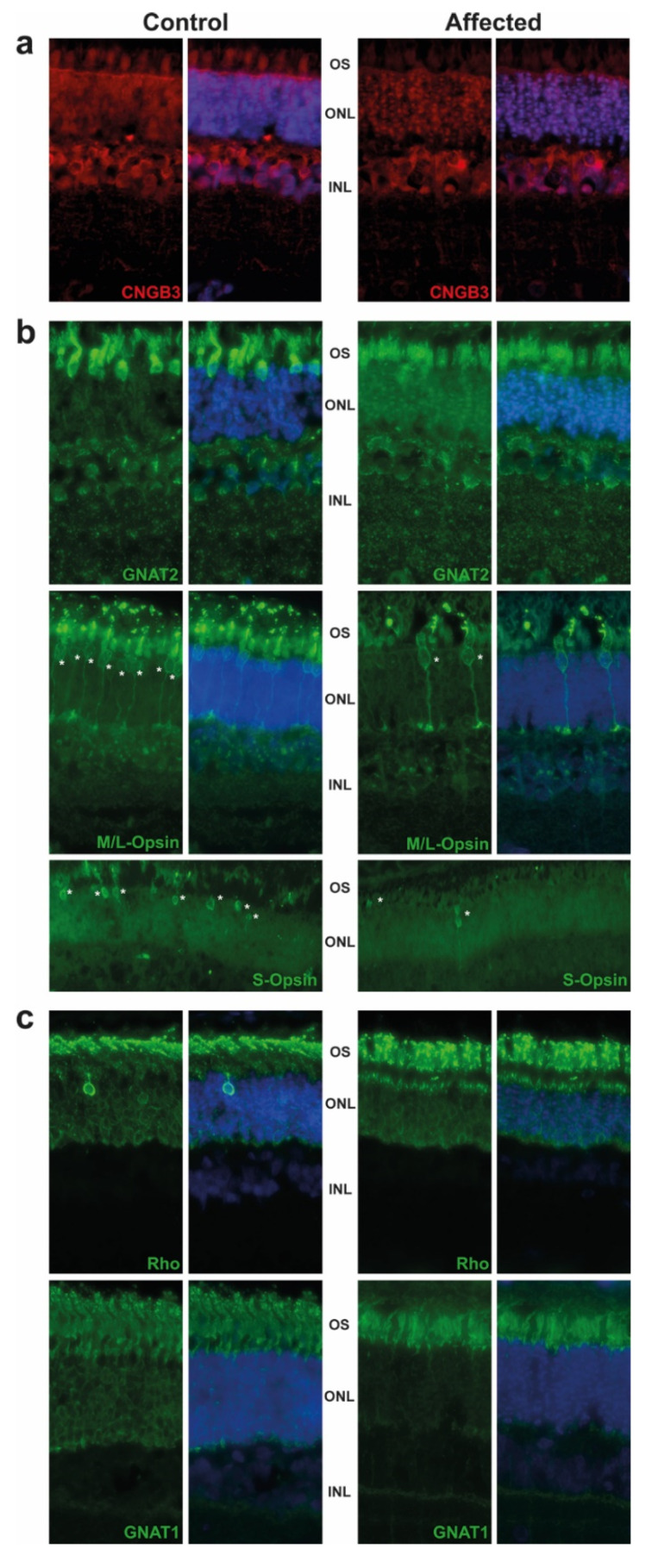
Immunostaining of retinal markers in a five-month old achromatopsia-affected cattle (case 12) and a control cattle of the same age. Cyclic nucleotide gated channel subunit beta 3 (CNGB3) (**a**), Cones markers (GNAT2, M/L-OPSIN, S-OPSIN) (**b**) and rods markers (GNAT1, RHODOPSIN) (**c**) are immunostained in both control and affected animals accordingly to conditions described in Table S2. Cell nuclei are shown in blue with DAPI. Images acquired at equal distances from the optic nerve head for each protein. Asterisks (*) show M/L- and S-Opsin cones present in both control and affected animal. Negative controls without primary antibody were performed (not shown).

The retina were targeted by immunofluorescence. We observed the expression of CNGB3 in the outer segment (OS) of cones in the control animal, while this expression was decreased in the affected animal. We also detected a significant difference of staining in the outer nuclear layer (ONL), where the CNGB3 protein seemed to be concentrated in the affected animal. This may suggest a mislocalization of the mutated CNGB3. Immunoreactivity of CNGB3 in the outer plexiform layer (OPL) may be due to an unspecific signal as the localization is observed in both control and affected animals. (Figure 2a). Further experiments on other affected calves (we analysed only one) or by transfection of the mutated CNGB3 in cells will confirm the potential higher stability of the mutated cyclic nucleotide-gated channel.

Based on GNAT2 and cone opsins staining, we clearly observed a decrease in the cone OS, which are shorter and abnormally shaped. Although we did not count the number of cones per retina, we observed a decrease in their number, in affected animal in comparison to control, as shown in M/L-and S-Opsin staining (Figure 2b, white asterisk). These results are in correlation with the ERG and are consistent with the progressive loss of cone outer segments seen in other species with achromatopsia. We also observed a slight decrease of the rod OS length in affected animals and a mislocalization of Rho (Figure 2c).

### 2.3. Pedigree Analysis

**Figure 3:**
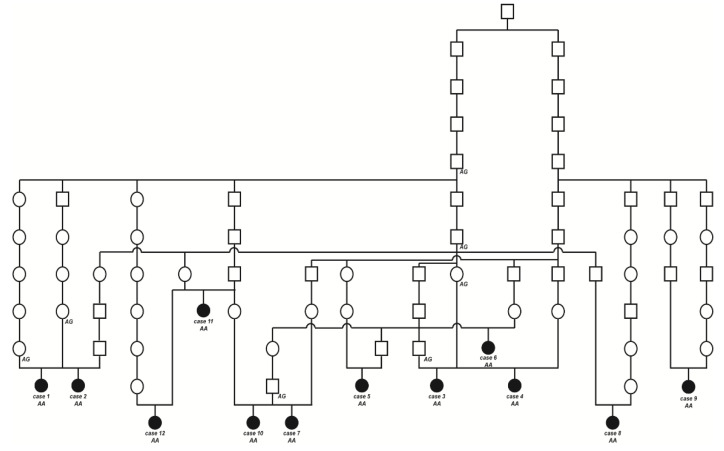
Pedigree of the Original Braunvieh cattle family segregating for achromatopsia suggested monogenic autosomal recessive inheritance. Affected animals are filled symbols. Open symbols represent normal cattle. DNA samples were available from animals with genotypes for the *CNGB3* XM_015474554.2:c.751G>A variant are given below the symbols.

The initially studied cases 1 and 2 were both the only affected animals in two different Swiss herds of purebred Original Braunvieh cattle. The sire of these two cases was a natural service purebred Original Braunvieh bull, which sired a further 24 apparently normal offspring within two years. A query to the Original Braunvieh breeders in Switzerland revealed further evidence of ten similar cases sired by different bulls collected over a period of three years. The available pedigree records of all 12 cases were analyzed and multiple inbreeding loops between the parents were found (Figure 3). We detected a single common ancestor occurring 8–11 generations ago. Due to the obvious history of inbreeding, a recessive inherited condition was considered. In light of the obvious consanguinity as well as the apparently unaffected parents, we hypothesize that the achromatopsia-affected calves might be explained by a recessively inherited variant. The founding mutation thus probably occurred many generations before the cases occurred. The causal variant was probably spread by the common ancestor, an artificial insemination bull born in 1961, as well as by some of his male descendants.

### 2.4. Genetic Analysis

**Figure 4:**
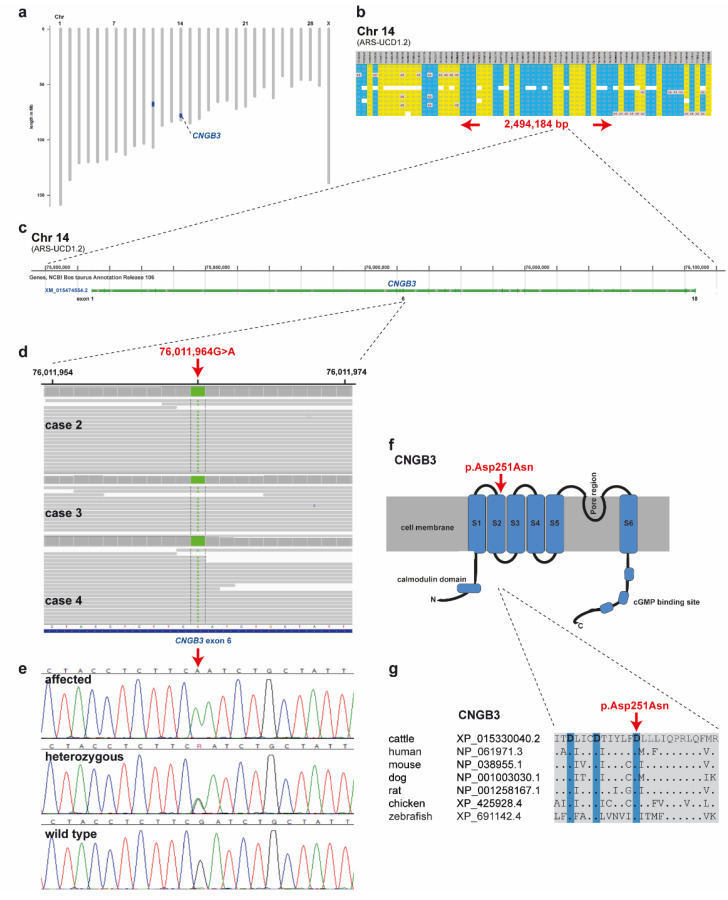
Achromatopsia-associated *CNGB3* missense variant in Original Braunvieh. (**a**) Genome-wide homozygosity mapping presenting the two homozygous blocks shared in 12 affected calves in blue. Note the red arrow highlighting the *CNGB3* gene on cattle chromosome 14. (**b**) Schematic representing the SNP genotypes of 12 affected calves on chromosome 14. Each horizontal lane represents one calf with yellow and blue shading, indicating shared homozygosity. Grey shading indicates a heterozygous genotype and white indicates missing genotypes. The genome positions of markers are indicated above the figure. The red arrows indicate the consensus homozygous region that spans approximately 2.5 Mb. (**c**) *CNGB3* gene structure showing the location of the exon 6 variant. (**d**) Genome viewer screenshot presenting the homozygous Chr14: g.76011964A>G variant in three affected calves. (**e**) Electropherograms showing the different genotypes identified via Sanger sequencing. (**f**) Localization of the missense variant (red arrow) with respect to the topological model of the CNGB3 protein. (**g**) Across species sequence alignment of the affected CNGB3 S2 domain. Note that the missense variant (red arrow) affects the evolutionary conserved Tri-Asp motif that is highlighted in blue.

SNP genotyping data for twelve affected cattle identified two shared ROHs between all cases on chromosome 11 and 14 (Figure 4a). On chromosome 11 all animals were homozygous for 101 SNP markers from 66,668,989 to 68,938,216 corresponding to a repeatedly detected strong selection signature of the Original Braunvieh breed encompassing a genome region with 24 protein-coding genes [15,16,17]. As no candidate gene for a retinopathy was contained in that region, we focused on the second ROH found on chromosome 14. All twelve affected cattle were homozygous at 28 SNP markers on chromosome 14 from 74,306,245 to 76,800,429, which allowed the identification of a single disease-associated IBD haplotype shared by all cases, limiting the critical region to 2,494,184 bp on chromosome 14 (Table S1; Figure 4a,b). Interestingly, the bovine homolog of *CNGB3*, a gene that causes achromatopsia in other species, maps to that genome region at 76 Mb (Figure 4c).

Subsequently, we sequenced the genomes of three of the affected cattle (cases 2, 3 and 4) and searched for private variants that were exclusively present in a homozygous state in all three affected cattle and absent or only heterozygous in the genomes of 567 other cattle. Beside 34 non-coding variants, all located in the critical region on chromosome 14, this analysis identified a single homozygous private protein-changing variant in *CNGB3*, a known candidate gene for achromatopsia. The variant can be designated as chr14: 76011964G>A (ARS-UCD1.2 assembly) (Figure 4d). It is a missense variant, XM_015474554.2: c.751G>A, predicted to change a highly conserved aspartic acid residue in the second S2 domain of CNGB3, XP_015330040.2: p.Asp251Asn (Figure 4f,g). In silico analysis predicted the functional effect of p.Asp251Asn as deleterious using PROVEAN software (score −4.985) [18].

We confirmed the presence of the *CNGB3* missense variant by Sanger sequencing (Figure 4e). The genotypes at the variant co-segregated with the achromatopsia phenotype as expected for a monogenic autosomal recessive mode of inheritance (Figure 3). All twelve available DNA samples from the achromatopsia-affected cattle carried the mutant allele in a homozygous state, while their parents were heterozygous, as expected for obligate carriers (Figure 3; Table 1).

We also genotyped the *CNGB3*: c.751G>A variant in a population control cohort comprising 2952 Original Braunvieh cattle without any phenotypic records. The mutant *CNGB3* allele was detected in the homozygous state in 12 of the cattle, whereas 463 were heterozygous carriers revealing an allele frequency of the mutant allele of 8.2% (Table 1). Interestingly, the mutant allele was absent from more than 35,000 cattle of various other breeds (Table 1). We found some rare heterozygous carriers with an allele frequency of 0.2% only in the Brown Swiss population of Switzerland (Table 1).

## 3. Discussion

To date, no genetic mutations have been associated with retinopathies in cattle. Affected Original Braunvieh calves with suspected vision disturbance suffer from day-blindness due to congenitally reduced cone-mediated function of the retina. Our clinicopathological evaluation of affected calves supported a diagnosis of achromatopsia based on abnormally appearing cone outer segments and normal rod photoreceptors. Furthermore, neither retinal degenerative changes nor abnormalities in the central visual pathways were observed. Pedigree and ROH analysis suggested an autosomal recessive mode of inheritance. Genome-wide homozygosity mapping using SNP array data was used successfully for high-resolution mapping of two critical regions of shared homozygosity. We performed whole-genome sequencing on three affected Original Braunvieh calves with day-blindness to identify variants associated with the phenotype. The similar clinical presentation between familial achromatopsia in humans and bovine recessive day-blindness led to the hypothesis that a protein-changing variant within *CNGA3*, *CNGB3*, *GNAT2*, *ATF6*, *PDE6C*, and *PDE6H* would be associated with achromatopsia of Original Braunvieh calves. Whereas only *CNGB3* was located in an IBD segment, we identified a missense variant in *CNGB3*: c.751G>A, p.Asp251Asn that significantly associated with the phenotype. Nine additional affected calves were subsequently genotyped and homozygous for the missense variant. Therefore, the cattle studied could serve as an animal model to further investigate the function of *CNGB3* in mammals.

In domestic animals, to this point in sheep [11,12,13] and dogs [19,20,21,22] (previously reported as cone degeneration and canine hemeralopia), the underlying genetics of different forms of achromatopsia are reported. Cones alone are affected in Alaskan Malamute (OMIA 001365-9615) and the German shorthaired pointers (OMIA 001676-9615) because of breed specific mutations in *CNGB3*, a cone–specific gene. Cone cyclic nucleotide-gated channels (CNG) are tetramers formed by three CNGA3 and one CNGB3 subunit; CNGA3 subunits can function as homotetrameric channels but CNGB3 exhibits channel function only when co-expressed with CNGA3 [23]. A 140-kb deletion and a missense mutation in *CNGB3* occurs in achromatopsia-affected dogs of multiple breeds [20]. Interestingly, the described canine missense variant also leads to an exchange of an aspartic acid with an asparagine residue, compromising a critical functional domain, and the phenotype seen in homozygous dogs represents a loss of function. Similar to what we found in Original Braunvieh cattle, the *CNGB3* missense mutation causing achromatopsia in German shorthaired pointers is also located in exon 6 (c.784G, p.Asp262Asn), affecting the corresponding residue of a conserved region of the same gene, suggesting an important role for this aspartate residue in channel biogenesis and/or function [21]. The flanking region surrounding these missense mutations is well conserved between species and is predicted to encode the second transmembrane domain of the CNGB3 protein containing three Asp residues designated the *tri-Asp motif* and conserved in all CNG channels [21]. Mutations of these conserved aspartate residues result in the absence of nucleotide-activated currents in heterologous expression. Aspartate is a negatively charged, polar amino acid found in both dogs and cattle with achromatopsia replaced by asparagine (Asn), another polar amino acid, which differs only in that it contains an amino group in place of one of the oxygens found in aspartate (Asp) and thus lacks a negative charge. Obviously, retinopathies associated with missense mutations draw attention to amino acids important for understanding the structure-function properties of functionally important channels. By in vitro follow-up studies of *CNGB3*-related canine achromatopsia it was found that Asp/Asn mutations affect the heteromeric subunit assembly of the six transmembrane-spanning helices (S1–S6), resulting in the loss of these inter-helical interactions altering the electrostatic equilibrium within in the S1–S4 bundle [21]. Although disease-causing variants within the S2 segment of human CNGB3 have not been reported (OMIM 605080), a study involving a missense mutation p.Asp211Glu at S2 of CNGA3 confirmed that variations in a conserved region could lead to cone dysfunction [24].

In Switzerland, the Original Braunvieh population is the ancestor of the world-renowned Brown Swiss population, which originated in North America from animals obtained in Switzerland at the turn of the century around 1900 [25]. Therefore, we speculate that the sporadic occurrence of *CNGB3*-carriers in the current Brown Swiss population indicates that the mutation might have arisen before that time and predates modern pedigree records. In recent decades, outbreaks of four undesirable genetic defects (weaver disease, spinal dysmyelination, spinal muscular atrophy, and arachnomelia) have occurred in Brown Swiss cattle. This report represents the first genetic disorder known in Original Braunvieh cattle which we have designated OH1, and the obtained results enable targeted selection to avoid the occurrence of further affected animals in future.

In summary, this study highlights the strong genetic similarities between human and bovine achromatopsia, suggesting that bovine achromatopsia, similar to that found in dogs, could serve as an excellent model for developing treatment strategies for humans.

## 4. Materials and Methods

### 4.1. Animal Selection for Genetic Analysis

This study was conducted with 248 Original Braunvieh cattle samples. The case cohort of this study consisted of twelve purebred Original Braunvieh cattle with suspected congenital vision disturbance reported to the breeding organization by different farmers between 2017 and 2020 (Table S1). In addition, either hair root or EDTA blood samples of three dams and two sires were collected for the genetic analysis, and genomic DNA was extracted using the Promega Maxwell^®^ RSC system (Promega, Dübendorf, Switzerland). The remaining 231 male Original Braunvieh cattle were used as population controls. These bulls had reliable phenotype records on normal vision because they very carefully examined by veterinarians before being used for artificial insemination. Before admission to the insemination station, these young bulls are carefully examined and these examinations include, in particular, the consideration of the presence of possible congenital disorders, including a standard ophthalmological examination of the eyes.

Once the most likely causative variant was discovered, it was added to two Swiss Axiom^®^ genotyping arrays (Thermo Fisher Scientific, Waltham, MA, USA) routinely used for genomic selection. Thus, after two years of population-wide genotyping in Swiss dairy populations for the purpose of genomic selection, more than 30,000 genotypes for the *CNGB3* variant were available. These were mainly determined in the four largest Swiss dairy cattle populations (Brown Swiss, Holstein, Original Braunvieh and Simmental).

### 4.2. Ophthalmological Examination including Electroretinography

A four-month-old calf (case 2) was presented to the University of Zürich Food Animal Clinic and the Ophthalmology Section in summer 2017 for evaluation of suspected vision disturbance.

A 5.5-month-old calf (case 12) was presented to the University of Bern Food Animal Clinic and the Division of Ophthalmology in the year 2020 for evaluation of suspected vision disturbance. Pupillary light responses were examined with bright red and blue light stimulation (Melan-100, 200–250 kcd, Iris-vet series, BioMed Vision Technologies, Ames, IA, USA). Furthermore, slit-lamp biomicroscopic examination (SL-17 Portable Slit Lamp, Kowa, Japan) of the adnexa and anterior segments, fluorescein staining (Contacare Ophthalmics and Diagnostics, Gujarat, India) of the corneas and indirect ophthalmoscopy (Omega 500 Binocular Indirect Ophthalmoscope, Heine Optotechnik GmBH, Gilching, Germany) of the ocular fundi were carried out. Intraocular pressures were measured by rebound tonometry (Tonovet Rebound Tonometer, Icare, Finland).

Electroretinograms (ERGs) were recorded under general anesthesia in case 12 following clinical and behavioural examinations. A jugular intravenous catheter was placed and the calf was sedated with xylazine 0.2 mg/kg IM. Induction was performed with ketamine 4 mg/kg intravenous, and 10 min after sedation the calf was placed in lateral recumbency. The head was positioned with cushions to facilitate access to the eye for testing. Anesthesia was maintained using ketamine continuous-rate infusion 3 mg/kg/h and xylazine continuous-rate infusion 0.05 mg/kg/hr. Flow-by oxygen was administered continuously via nasal oxygen catheter. All recordings were conducted on the right eye, following dilation of the pupil with 1% tropicamide ophthalmic solution. ERGs were recorded using the RetiPORT ERG system (Roland Consult, Brandenburg an der Havel, Germany). Two platinum subdermal needle electrodes (Grass Safelead Needle electrodes, Grass Technologies, West Warwick, RI, USA) were used: The reference electrode was placed subcutaneously approximately 10 mm from the lateral canthus, and the ground electrode was placed over the occipital protuberance. An ERG-Jet^®^ corneal electrode (Fabrinal SA, La Chaux-de-Fonds, Switzerland) was used as the active electrode and applied with 2.5% hypromellose ophthalmic demulcent solution. Flash stimuli and light adaptation were delivered using a handheld Mini Ganzfeld (Roland Consult).

Following 20 min of dark adaptation, mixed cone-rod responses were recorded with a flash intensity of 0.096 cd.s/m^2^ (average of 3 sweeps at 0.1 Hz). Subsequently, the eye was light-adapted for 5 min to a white uniform background light of 30 cd/m^2^ and single cone (average of 3 sweeps at 1.0 Hz) and cone flicker (average of 3 sweeps at 28 Hz) responses were recorded with a 3.0 cd.s/m^2^ flash intensity. For all recordings, the filters were set to allow a bandpass of 1 to 300 Hz.

### 4.3. Targeting Cones by Immunofluorescence

After slaughtering, the enucleated calf eyes of case 12 were fixed in Bouin’s solution for 24 h, trimmed and paraffin embedded. The 3 µm-embedded paraffin sections were further processed for immunofluorescence. Briefly, retina sections were first deparaffinized by successive baths (three different Xylol baths of 5 min, 3 min, 3 min respectively; and six ethanol baths: from 100% to 70%; then washed several times in water). Sections were then boiled 30 min in a Dako antigen retrieval solution (Agilent S169984-2) and left to cool down for 45 min. Retina sections were incubated for 1 h in blocking solution and incubated with primary antibodies as indicated in Table S2. Following incubation with primary antibodies, sections were washed 3 times in PBS and incubated for 1.5 h at RT with the secondary antibodies (Table S2). After three successive washing steps in PBS, sections were treated for 25 min in 0.1% Sudan black B (Sigma 380B)/70% ethanol. Then, sections were washed again twice in ethanol 70%, and three times in PBS 0.02% Tween and counterstained with 49,6-Diamidino-2-phenylindole (DAPI) to identify retinal cell layers. After three washing steps in PBS, sections were mounted with antifadent citifluor solution (Electron microscopy sciences, Hatfield, PA, USA). Immunostaining was visualized under a fluorescence microscope (Leica, Switzerland). Incubation with the secondary antibody alone was used as a negative control, and every image acquisition of the retina was made at the same distance from the optic nerve head for each antigen.

### 4.4. Morphology and Histopathology of the Visual Pathway

The head of case 1 was taken for gross and histopathological evaluation after slaughtering at the age of five months. The visual pathways of the affected cattle were evaluated and biopsies fixed in 4% formaldehyde for routine histopathological evaluation with haematoxylin and eosin (H&E) staining of the optic nerve, optic tract, optic chiasm, lateral geniculate nucleus and the visual cortex.

### 4.5. SNP Genotyping and Subsequent Homozygosity Mapping with 12 Affected Cattle

Genotype data for the twelve achromatopsia-affected cattle (cases 1–12) were obtained with an Illumina BovineHD BeadChip array. The PLINK v1.9 software [26] was used to perform basic quality filtering of the dataset. For homozygosity mapping, the genotype data for the twelve affected cattle were used. Markers on the sex chromosomes were excluded. The following PLINK option parameters were applied (--homozyg-snp 10; --homozyg group; --homozyg-density 30; --homozyg-gab 1000; --homozyg-window-het 0; --homozyg-window-missing 0) to search for extended regions of homozygosity (ROH) indicating chromosomal region of identity-by-descent (IBD). ROH analyses were performed using an imputed dataset that included the entire Swiss genotype archive for Original Braunvieh. Animals were genotyped using several routinely available array chips that included between nine and 777 k SNPs. The available genotype archive was used in a two-step imputation approach and was imputed first to a density of 150 k. Subsequently, imputation to 777 k-density was carried out using 150 k data. A number of 2507 and 351 Original Braunvieh reference animals were available for 150 k and 777 k imputation, respectively. FImpute v2.2 software was used with default parameters for both steps [27]. In each step, SNPs with a minor allele frequency (MAF) lower than 1% were removed from the dataset. The final marker set included 114891 and 681179 SNPs for each density (150 k and 777 k), respectively. SNPs were filtered using the following thresholds: MAF higher than 0.01 and an SNP call rate higher than 0.99 in the genotype data from the reference population. The output interval was displayed in Excel spreadsheets to find overlapping regions (Table S1). All positions correspond to the ARS-UCD1.2 reference genome assembly.

### 4.6. Whole-Genome Resequencing and Variant Calling

Three Illumina TruSeq PCR-free libraries with ~500 bp insert size were prepared from three affected cattle (cases 2, 3 and 4). We collected 2 × 150 bp paired-end reads on a NovaSeq 6000 instrument. Mapping to ARS-UCD1.2 reference genome assembly was performed as described [28]. The sequence data were deposited under study accession PRJEB28191 and sample accessions SAMEA4644768, SAMEA6528889 and SAMEA6528891 at the European Nucleotide Archive.

Variant calling including single-nucleotide variants (SNVs) and small indels was performed as described [29]. To predict the functional effects of the called variants, SnpEff software v4.3 [30] together with the ARS-UCD1.2 reference genome assembly and NCBI Annotation Release 106 (https://www.ncbi.nlm.nih.gov/genome/annotation_euk/Bos_taurus/106/; accessed on 30 June 2021) was used. For private variant filtering we used control genome sequences from 567 cattle of diverse breeds including 119 Original Braunvieh animals. These genomes were produced during the Swiss Comparative Bovine Resequencing project and made publicly available (https://www.ebi.ac.uk/ena/browser/view/PRJEB18113/; accessed on 30 June 2021). The most likely pathogenic missense variant in *CNGB3* was inspected for its presence in a global control cohort of 3306 genomes with a sequence depth of at least 8x from a variety of breeds including 92 Original Braunvieh animals (1000 Bull Genomes Project run 8; www.1000bullgenomes.com accessed on 30 June 2021) [29].

### 4.7. Genotyping Assays

Two genotyping tests were developed for the XM_015474554.2:c.751G > A missense variant in the *CNGB3* gene to confirm segregation with disease and to estimate the allele frequency in the population.

#### 4.7.1. PCR and Sanger Sequencing

We designed a specific PCR for the targeted genotyping of the chr14:76011964G>A variant. PCR was performed for 30 cycles using Amplitaq Gold Master Mix (Thermofisher, Rotkreuz, Switzerland) in a 10 μL reaction containing 10 ng genomic DNA and 5 pmol of each primer (F 5′-CCTGTGGCTCTCACTTGTCA-3′ and R 5′- CTCCCGAGCCCCTACTTACT-3′). After treatment with exonuclease I and alkaline phosphatase, PCR amplicons were sequenced on an ABI 3730 DNA Analyzer (Thermofisher, Rotkreuz, Switzerland). Sanger sequences were analyzed using the Sequencher 5.1 software (GeneCodes, Ann Arbor, MI, USA).

#### 4.7.2. Axiom^®^ Genotyping Array

Two fully customized Axiom^®^ genotyping arrays (Thermo Fisher Scientific, Rotkreuz, Switzerland) designed for genomic selection purpose in Swiss dairy cattle populations designated as SWISScow (96-array layout with 314,744 markers) and SWISSLD1 (384-array layout with 64,212 markers) both included the chr14:76011964G>A variant.

## Figures and Tables

**Table 1:** Association of the missense variant in *CNGB3* with the achromatopsia phenotype in Original Braunvieh cattle.

	GG	AG	AA
Achromatopsia-affected calves			12
Obligate carriers ^a^		5	
Other Original Braunvieh cattle ^b,c^	2477	463	12
Brown Swiss cattle ^c^	14,976	52	
Holstein cattle ^c^	14,825		
Simmental cattle ^c^	2021		
Sequenced cattle genomes from various breeds (local Swiss cohort) ^d^	552	15	
Control cattle from various breeds (1000 Bull Genomes project) ^e^	3298	7 ^f^	1 ^g^

^a^ parents of affected animals were classified as obligate carriers. ^b^ phenotypes are unknown. ^c^ Axiom^®^ genotype data from population-wide routine genomic testing. ^d^ 567 genomes of the Swiss Comparative Bovine Resequencing project including 92 Original Braunvieh cattle. ^e^ run 8: 3306 genomes including 58 Original Braunvieh cattle. ^f^ exclusively Original Braunvieh. ^g^ case 2 was added to the 1000 Bull Genomes project.

## Data Availability

The WGS data of the three sequenced cases can be found in the European Nucleotide Archive under the sample accession nos. SAMEA4644768 (case 2), SAMEA6528889 (case 3), and SAMEA6528891 (case 4).

## Supplementary Materials

The following are available online at https://www.mdpi.com/article/10.3390/ijms222212440/s1.

## References

[1] Lavach J.D. (1990). Large Animal Ophthalmology.

[2] Williams D.L. (2010). Production animal ophthalmology. Vet. Clin. N. Am. Food Anim. Pract..

[3] Williams D.L. (2010). Welfare issues in farm animal ophthalmology. Vet. Clin. N. Am. Food Anim. Pract..

[4] Leipold H.W. (1984). Congenital ocular defects in food-producing animals. Vet. Clin. N. Am. Large Anim. Pract..

[5] Gelatt K.N., Ben-Shlomo G., Gilger B.C., Hendrix D.V., Kern T.J., Plummer C.E. (2021). Veterinary Ophthalmology.

[6] Williams D.L. (2010). Congenital abnormalities in production animals. Vet. Clin. N. Am. Food Anim. Pract..

[7] He X., Li Y., Li M., Jia G., Dong H., Zhang Y., He C., Wang C., Deng L., Yang Y. (2012). Hypovitaminosis A coupled to secondary bacterial infection in beef cattle. BMC Vet. Res..

[8] Murgiano L., Jagannathan V., Calderoni V., Joechler M., Gentile A., Drögemüller C. (2014). Looking the cow in the eye: Deletion in the NID1 gene is associated with recessive inherited cataract in Romagnola cattle. PLoS ONE.

[9] Hollmann A.K., Dammann I., Wemheuer W.M., Wemheuer W.E., Chilla A., Tipold A., Schulz-Schaeffer W.J., Beck J., Schütz E., Brenig B. (2017). Morgagnian cataract resulting from a naturally occurring nonsense mutation elucidates a role of CPAMD8 in mammalian lens development. PLoS ONE.

[10] Sun W., Li S., Xiao X., Wang P., Zhang Q. (2020). Genotypes and phenotypes of genes associated with achromatopsia: A reference for clinical genetic testing. Mol. Vis..

[11] Reicher S., Seroussi E., Gootwine E. (2010). A mutation in gene CNGA3 is associated with day blindness in sheep. Genomics.

[12] Ross M., Ofri R., Aizenberg I., Abu-Siam M., Pe’er O., Arad D., Rosov A., Gootwine E., Dvir H., Honig H. (2020). Naturally-occurring myopia and loss of cone function in a sheep model of achromatopsia. Sci. Rep..

[13] Komáromy A.M. (2010). Day blind sheep and the importance of large animal disease models. Vet. J..

[14] Winkler P.A., Occelli L.M., Petersen-Jones S.M. (2020). Large animal models of inherited retinal degenerations: A review. Cells.

[15] Rothammer S., Seichter D., Förster M., Medugorac I. (2013). A genome-wide scan for signatures of differential artificial selection in ten cattle breeds. BMC Genom..

[16] Signer-Hasler H., Burren A., Neuditschko M., Frischknecht M., Garrick D., Stricker C., Gredler B., Bapst B., Flury C. (2017). Population structure and genomic inbreeding in nine Swiss dairy cattle populations. Genet. Sel. Evol..

[17] Bhati M., Kadri N.K., Crysnanto D., Pausch H. (2020). Assessing genomic diversity and signatures of selection in Original Braunvieh cattle using whole-genome sequencing data. BMC Genom..

[18] Choi Y., Chan A.P. (2015). PROVEAN web server: A tool to predict the functional effect of amino acid substitutions and indels. Bioinformatics.

[19] Miyadera K., Acland G.M., Aguirre G.D. (2012). Genetic and phenotypic variations of inherited retinal diseases in dogs: The power of within- and across-breed studies. Mamm. Genome.

[20] Yeh C.Y., Goldstein O., Kukekova A.V., Holley D., Knollinger A.M., Huson H.J., Pearce-Kelling S.E., Acland G.M., Komáromy A.M. (2013). Genomic deletion of CNGB3 is identical by descent in multiple canine breeds and causes achromatopsia. BMC Genet..

[21] Tanaka N., Delemotte L., Klein M.L., Komáromy A.M., Tanaka J.C. (2014). A cyclic nucleotide-gated channel mutation associated with canine daylight blindness provides insight into a role for the S2 segment tri-Asp motif in channel biogenesis. PLoS ONE.

[22] Tanaka N., Dutrow E.V., Miyadera K., Delemotte L., MacDermaid C.M., Reinstein S.L., Crumley W.R., Dixon C.J., Casal M.L., Klein M.L. (2015). Canine CNGA3 Gene Mutations Provide Novel Insights into Human Achromatopsia-Associated Channelopathies and Treatment. PLoS ONE.

[23] Sidjanin D.J., Lowe J.K., McElwee J.L., Milne B.S., Phippen T.M., Sargan D.R., Aguirre G.D., Acland G.M., Ostrander E.A. (2002). Canine *CNGB3* mutations establish cone degeneration as orthologous to the human achromatopsia locus ACHM3. Hum. Mol. Genet..

[24] Chen X.T., Huang H., Chen Y.H., Dong L.J., Li X.R., Zhang X.M. (2015). Achromatopsia caused by novel missense mutations in the CNGA3 gene. Int. J. Ophthalmol..

[25] Hagger C. (2005). Estimates of genetic diversity in the brown cattle population of Switzerland obtained from pedigree information. J. Anim. Breed. Genet..

[26] Purcell S., Neale B., Todd-Brown K., Thomas L., Ferreira M.A.R., Bender D., Maller J., Sklar P., De Bakker P.I.W., Daly M.J. (2007). PLINK: A tool set for whole-genome association and population-based linkage analyses. Am. J. Hum. Genet..

[27] Sargolzaei M., Chesnais J.P., Schenkel F.S. (2011). FImpute—An efficient imputa- tion algorithm for dairy cattle populations. J. Dairy Sci..

[28] Häfliger I.M., Wiedemar N., Švara T., Starič J., Cociancich V., Šest K., Gombač M., Paller T., Agerholm J.S., Drögemüller C. (2020). Identification of small and large genomic candidate variants in bovine pulmonary hypoplasia and anasarca syndrome. Anim. Genet..

[29] Hayes B.J., Daetwyler H.D. (2019). 1000 Bull Genomes Project to Map Simple and Complex Genetic Traits in Cattle: Applications and Outcomes. Annu. Rev. Anim. Biosci..

[30] Cingolani P., Platts A., Wang L.L., Coon M., Nguyen T., Wang L., Land S.J., Lu X., Ruden D.M. (2012). A program for annotating and predicting the effects of single nucleotide polymorphisms, SnpEff: SNPs in the genome of Drosophila melanogaster strain w1118; iso-2; iso-3. Fly.

