# Supplementary material for: CNGB3 Missense Variant Causes Recessive Achromatopsia in Original Braunvieh Cattle
Source: Int J Mol Sci. 2021 Nov 18;22(22):12440. doi: 10.3390/ijms222212440 (PMC8620519; doi:10.3390/ijms222212440)
Supplement: Supplementary file 1 [file ijms-22-12440-s001.zip › FigureS1.pdf]

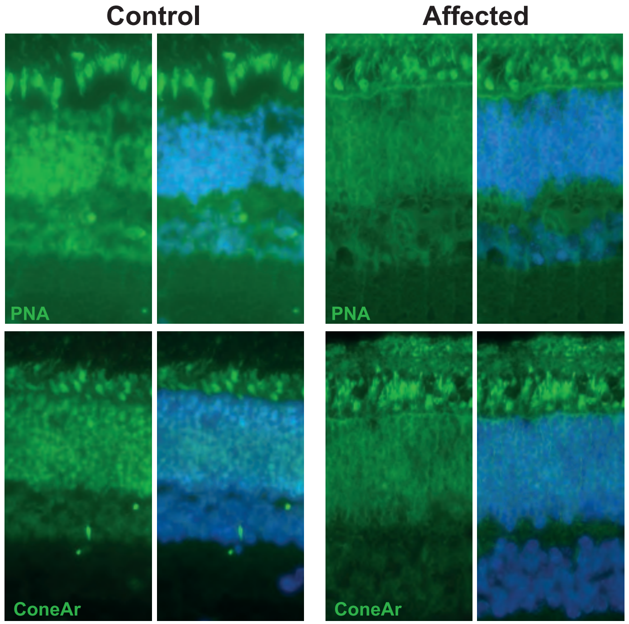

Figure S1: Immunostaining of cones markers in control and affected calf. PNA and CONE AR-RESTIN (ConeAr) are immunostained in both control and affected animals accordingly to condi-tions described in Table S2.
