# Supplementary material for: CNGB3 Missense Variant Causes Recessive Achromatopsia in Original Braunvieh Cattle
Source: Int J Mol Sci. 2021 Nov 18;22(22):12440. doi: 10.3390/ijms222212440 (PMC8620519; doi:10.3390/ijms222212440)
Supplement: Supplementary file 1 [file ijms-22-12440-s001.zip › TableS2.pdf]

**Table S2:** References of antibodies and immunostaining conditions used to detect retinal markers in a 5.5-month old achromatopsia-affected calf (case 12) and a control cattle.

| Antibody | Reference | Species | Blocking condition | Dilution |
|---|---|---|---|---|
| CNGB3 | Biorbyt ORB658475 | Rabbit | 10% NGS, 0.3% Triton / PBS ON at 4°C | 1/500 |
| GNAT2 | SantaCruz SC-390 | Rabbit | 10% NGS, 0.3% Triton / PBS ON at 4°C | 1/200 |
| ML-OPSIN | Sigma AB5405 | Rabbit | 10% NGS, 0.3% Triton / PBS ON at 4°C | 1/2000 |
| S-OPSIN | SantaCruz SC-14363 | Goat | 5% NDS, 0.1% Triton / PBS ON at 4°C | 1/1000 |
| RHODOPSIN (RET-P1) | Thermofisher MS-1233-P | Mouse | 10% NGS, 0.3% Triton / PBS ON at 4°C | 1/500 |
| GNAT1 | SantaCruz SC-389 | Rabbit | 10% NGS, 0.3% Triton / PBS ON at 4°C | 1/1000 |
| PNA-FITC | Sigma L3766 | - | 10% NGS, 0.3% Triton / PBS ON at 4°C | 1/1000 |
| CONE ARRESTIN | Sigma 15282 | Rabbit | 10% NGS, 0.3% Triton / PBS ON at 4°C | 1/10000 |
| Anti-rabbit AlexaFluor 488 | Invitrogen A11070 | Goat | PBS 1h30 RT | 1/2000 |
| Anti-mouse AlexaFluor 488 | Invitrogen A21121 | Goat | PBS 1h30 RT | 1/2000 |
| Anti-goat AlexaFluor 488 | Invitrogen A11055 | Donkey | PBS 1h30 RT | 1/2000 |
